# Integrative genetic and single cell RNA sequencing analysis provides new clues to the amyotrophic lateral sclerosis neurodegeneration

**DOI:** 10.3389/fnins.2023.1116087

**Published:** 2023-02-17

**Authors:** Hankui Liu, Liping Guan, Min Deng, Lars Bolund, Karsten Kristiansen, Jianguo Zhang, Yonglun Luo, Zhanchi Zhang

**Affiliations:** ^1^Hebei Industrial Technology Research Institute of Genomics in Maternal and Child Health, BGI-Shijiazhuang Medical Laboratory, Shijiazhuang, China; ^2^BGI-Shenzhen, Shenzhen, China; ^3^Laboratory of Genomics and Molecular Biomedicine, Department of Biology, University of Copenhagen, Copenhagen, Denmark; ^4^Institute of Medical Innovation and Research, Peking University Third Hospital, Beijing, China; ^5^Lars Bolund Institute of Regenerative Medicine, BGI-Qingdao, Qingdao, China; ^6^Department of Biomedicine, Aarhus University, Aarhus, Denmark; ^7^Department of Human Anatomy, Hebei Medical University, Shijiazhuang, Hebei, China; ^8^Hebei Key Laboratory of Neurodegenerative Disease Mechanism, Hebei Medical University, Shijiazhuang, China

**Keywords:** amyotrophic lateral sclerosis (ALS), motor neurons (MNs), single-cell transcriptome, genome-wide association studies (GWAS), neurological disorder

## Abstract

**Introduction:**

The gradual loss of motor neurons (MNs) in the brain and spinal cord is a hallmark of amyotrophic lateral sclerosis (ALS), but the mechanisms underlying neurodegeneration in ALS are still not fully understood.

**Methods:**

Based on 75 ALS-pathogenicity/susceptibility genes and large-scale single-cell transcriptomes of human/mouse brain/spinal cord/muscle tissues, we performed an expression enrichment analysis to identify cells involved in ALS pathogenesis. Subsequently, we created a strictness measure to estimate the dosage requirement of ALS-related genes in linked cell types.

**Results:**

Remarkably, expression enrichment analysis showed that α- and γ-MNs, respectively, are associated with ALS-susceptibility genes and ALS-pathogenicity genes, revealing differences in biological processes between sporadic and familial ALS. In MNs, ALS-susceptibility genes exhibited high strictness, as well as the ALS-pathogenicity genes with known loss of function mechanism, indicating the main characteristic of ALS-susceptibility genes is dosage-sensitive and the loss of function mechanism of these genes may involve in sporadic ALS. In contrast, ALS-pathogenicity genes with gain of function mechanism exhibited low strictness. The significant difference of strictness between loss of function genes and gain of function genes provided a priori understanding for the pathogenesis of novel genes without an animal model. Besides MNs, we observed no statistical evidence for an association between muscle cells and ALS-related genes. This result may provide insight into the etiology that ALS is not within the domain of neuromuscular diseases. Moreover, we showed several cell types linked to other neurological diseases [i.e., spinocerebellar ataxia (SA), hereditary motor neuropathies (HMN)] and neuromuscular diseases [i.e. hereditary spastic paraplegia (SPG), spinal muscular atrophy (SMA)], including an association between Purkinje cells in brain and SA, an association between α-MNs in spinal cord and SA, an association between smooth muscle cells and SA, an association between oligodendrocyte and HMN, a suggestive association between γ-MNs and HMN, a suggestive association between mature skeletal muscle and HMN, an association between oligodendrocyte in brain and SPG, and no statistical evidence for an association between cell type and SMA.

**Discussion:**

These cellular similarities and differences deepened our understanding of the heterogeneous cellular basis of ALS, SA, HMN, SPG, and SMA.

## Introduction

Amyotrophic lateral sclerosis (ALS) is a neurodegenerative disease characterized by the progressive loss of motor neurons (MNs) in the brain and spinal cord (Kiernan et al., 2011). The incidence of ALS has been estimated to 1.65 per 100,000 people-year in China (Xu et al., 2020), 1.5 in United States (Mehta et al., 2022), and 3.19 in European (Marin et al., 2014). Genetics is a critical factor for ALS. In familial ALS, pathogenic mutations can be identified in about 60–80% of patients, of which *C9orf72* (40%), *SOD1* (20%), *FUS* (1–5%), and *TARDBP* (1–5%) are the most common (Renton et al., 2014). In sporadic ALS, the genetic contribution is estimated to 61% (95% CI: 38–78%) (Graham et al., 1997; Al-Chalabi et al., 2010). Large-scale genome-wide association studies (GWAS) have identified several susceptibility genes, including *C9orf72*, *SOD1*, and *UNC13A*. The ALS-related genes were implicated in altered protein homeostasis (*SOD1*), depositions of intranuclear RNA (*C9orf72*), and altered neuronal cytoskeletal dynamics (*TUBA4A*), leading to the death of upper and/or lower MNs in the brain, brainstem, and spinal cord (Brown and Al-Chalabi, 2017).

Although MN functions have been implicated, the mechanisms of neurodegeneration in ALS are still not fully understood (van Es et al., 2017). Moreover, the roles of two distinct subpopulations—α-MNs and γ-MNs—in ALS pathogenesis are not fully elucidated. α-MNs located in the spinal cord innervate extrafusal muscle fibers, which create force to move the skeleton. In contrast, γ-MNs innervate intrafusal fibers, which modulate the sensitivity of muscle spindles to stretch (Burke et al., 1977). A comparison of α- and γ-MNs in the spinal cord showed different behavior in ALS (Ragagnin et al., 2019), indicating that these two MNs subpopulations are not affected equally in ALS pathogenesis, raising the question if genetics contributes to the different roles of MNs subpopulations in ALS. Although several genes were found to be associated with familial and sporadic ALS, few studies have addressed possible genetic differences distinguishing familial and sporadic ALS and the cellular basis governing pathogenicity and susceptibility of ALS.

Recent advances of single-cell RNA-sequencing have provided an accurate and comprehensive depiction of cell types and gene expression. On the basis of cell type identification and diseases-related gene expression, previous studies (Skene et al., 2018; Bryois et al., 2020) have revealed various brain cell types involved in neurological disorders, including associations between monoaminergic neurons and neurodegenerative diseases, associations between embryonic GABAergic neurons and neurodevelopmental diseases, and associations between projecting excitatory neurons and psychotic disorders. Our previous study also showed that serotonergic neurons are involved in the etiology and therapy-genetics of anxiety disorders (Liu et al., 2021).

Here, using the same strategy, we applied an empirical method named expression weighted cell type enrichment (Skene and Grant, 2016) (EWCE) to investigate the cellular basis of ALS and four neurological disorders with phenotypic overlap, including hereditary motor neuropathies (HMN), spinocerebellar ataxia (SA), hereditary spastic paraplegia (SPG), spinal muscular atrophy (SMA). Our results suggested that MNs in the spinal cord play a role in ALS, SA, and HMN; additionally, MNs subpopulations—α- and γ-MNs—are mainly linked to the susceptibility and pathogenicity of ALS.

## Materials and methods

### Pathogenicity genes of ALS, HNM, SA, SPG, and SMA

We scanned the OMIM (Amberger et al., 2015) database (updated till July 14th, 2021) using the phenotypic series “PS105400-Amyotrophic lateral sclerosis” and discovered 37 causal connections between pathogenic mutations in genes and ALS subtypes (e.g., ALS 6, 8, 9, 10, 11, 12, 15, 18, 19, 20, 21, 22, 23, and 26, with or without frontotemporal dementia). We identified 32 unique pathogenicity genes and employed the gene symbols reported in OMIM (Supplementary Table 1). We searched the OMIM (Amberger et al., 2015) database (updated till July 14th, 2021) for the phenotypic series “PS164400-Spinocerebellar ataxia,” “PS303350-Spastic paraplegia,” as well as phenotypic description including of “hereditary motor neuropathies” and “spinal muscular atrophy” that do not belong to a single phenotypic series. Finally, we discovered 36, 15, 20, 57 genes that are related to HMN, SA, SMA, and SPG, respectively (Supplementary Table 2).

### Susceptibility genes of ALS

We collected 48 ALS-susceptibility genes from 13 recent large-scale genome-wide association studies (Laaksovirta et al., 2010; Shatunov et al., 2010; Deng et al., 2013; Fogh et al., 2014; Xie et al., 2014; van Rheenen et al., 2016, 2021; Benyamin et al., 2017; Nicolas et al., 2018; Wei et al., 2019; Iacoangeli et al., 2020; Nakamura et al., 2020; Li et al., 2021). We reviewed these studies carefully and employed the genes that were reported to be significantly associated with ALS (Supplementary Table 3) in single-variant association and/or gene-level association.

### Single-cell transcriptome datasets

We employed six large-scale single-cell transcriptome datasets from recent studies (Zeisel et al., 2015; Rosenberg et al., 2018; De Micheli et al., 2020; Supplementary Table 4). The first dataset was generated by Rosenberg et al. (2018). Their study analyzed 156,049 mononuclear transcriptomes in mouse brains and spinal cords and classified these cells into 73 cell types *via* an unsupervised clustering method. The second dataset was generated by De Micheli et al. (2020). The research team integrated 22,058 single-cell transcriptomes in human muscles (e.g., right serratus, left flexor hallucis longus, orbicularis oris, eye lid, left vastus lateralis, left external oblique, left rectus abdominus, trapezius, right external oblique, right flexor hallucis longus) and resolved 16 distinct populations of muscle-resident cells. The third dataset was generated by Milich et al. (2021). Their study generated 66,178 cells from uninjured and injured (Briefly, mice were anesthetized and received a 65-kilodyne mid-thoracic contusion injury) mouse spinal cord. Cluster analysis of these cells resulted in 15 distinct clusters. The fourth dataset was generated by Sathyamurthy et al. (2018). Their study analyzed 17,354 nuclei from adult mouse lumbar spinal cord and founded seven major clusters. The fifth dataset was generated by Andersen et al. (2021). Their study obtained transcriptomes of 112,554 cells and 34,884 nuclei from four samples of human spinal cord and indicated the cellular landscape of the human spinal cord, including α and γ MNs. The sixth dataset with 9,970 cells was assembled by Skene et al. (2018) from single-cell transcriptome datasets (Dueck et al., 2015; Saraiva et al., 2015; Usoskin et al., 2015; Zeisel et al., 2015). These cells are distributed in various mouse brain regions and were classified into 24 brain cell types. These datasets were released by the authors and accessed from the Gene Expression Omnibus database.

Datasets 1–2 containing single-cell transcriptomes of mouse brain, mouse spinal cord, and human muscle were used for discovery; Datasets 3–4 were used for replicating the associations that were founded in mouse spinal cord. Dataset 5 is used for validating the associations of mouse in human spinal cord. The reason we did not used it for discovery is that the dataset 5 is provided by a preprint study. Dataset 6 was used for validating the associations that were found in mouse brain. Since no cell type annotations of MNs and subpopulations were included in the datasets 3–4, we annotated MNs and subpopulations *via* the SingleR (Aran et al., 2019) R-package^1^ with reference to the spinal cord (Rosenberg et al., 2018) (dataset 1).

### Expression weighted cell type enrichment

The EWCE (Skene and Grant, 2016) method has been demonstrated to be reliable for studying the expression specificity across various cell types with single-cell transcriptomes. We employed the EWCE R-package^2^ to investigate the cell-type expression specificity of ALS-related genes. Firstly, we used the generate-celltype-data function to calculate the specificity of genes in each cell type. Subsequently, we used the bootstrap-enrichment-test function to estimate the *P*-value of specificity of target genes. The bootstrap method randomly samples 10,000 lists of genes with the same number of target genes from all the genes. The specificity of these 10,000 lists of genes was used as background distribution. *P*-values of specificity of target genes were calculated by the cumulative density function of the specificity distribution and adjusted by the false discovery rate (FDR) method. Statistical significances differed by genes number were estimated by randomly selecting the genes number (5–32) from ALS-pathogenicity genes and exhibited in Supplementary Table 5.

### Dosage requirement in related cells

We developed a strictness measure in this study to estimate the dosage requirement for a given gene. Strictness was calculated by the standard deviation of fold change: $S=1/\sqrt{\frac{\sum_{i}^{n}{\left(C_{i}-\bar{C}\right)}^{2}}{n-1}}$, in which *S* refers to strictness, *C* refers to fold change, *i* refers to the *i*th cell, and *n* refers to the total number of cells. The fold change was calculated by the expression in one cell divided by the mean expression in all cells: $C_{i}\,E_{i}/\bar{E}$, in which *E* refers to the expression in one cell, $\bar{E}$ refers to the mean expression, *i* refers to the *i*th cell. A high strictness value indicates that a gene is required to have strict expression. A low strictness indicates that the gene expression is tolerant to alterations.

To calculate the significance of strictness for target genes, we developed a method based on the Central Limit Theorem. Central Limit Theorem states that the distribution of the sample means will be approximately normal distribution. Firstly, we have a number (*n*) of target genes that we want to study, and then we calculate the strictness mean for these *n* genes (*x*). Subsequently, we take a sample with *n* genes from all the genes randomly and repeat the sampling 10, 000 times *via* a bootstrap method. We calculate the strictness means for each of the 10,000 random samples and then estimate the mean (μ) and the standard deviation (σ) of the distribution *via* a maximum likelihood estimation. The distribution of sample means should be approximately normal: *XN*(μ,σ^2^). Finally, the *P*-value of the target genes mean is calculated as: $P\,\left(x\,X\right)=1-\frac{1}{\sigma \,\sqrt{2\,\pi }}\,\int_{-\infty }^{x}-\text{exp}\,\left\{-\frac{{\left(x-\mu \right)}^{2}}{2\,\sigma^{2}}\right\}\,d\,x$. *P*-values are adjusted by the Bonferroni’s method. We employed the three gene sets described below for evaluating the performance of the strictness measure.

### Haploinsufficient genes

We accessed 299 known haploinsufficient (HI) genes from Dang et al. (2008). We retained 49 HI genes that are related to neurodegenerative and/or neurodevelopmental diseases (i.e., Alzheimer’s disease, Parkinson’s disease, Huntington’s disease, SA, multiple system atrophy, epilepsy, autism spectrum disorder, and schizophrenia) (Supplementary Table 6). These 49 genes were used as a positive control for evaluating the strictness measure.

### Loss-of-function tolerant genes

We accessed 330 putative homozygous loss-of-function tolerant (LoFT) genes from Lek et al. (2016) (Supplementary Table 6). These genes contain at least two different high confidence loss-of-function (LoF) variants that were found in a homozygous state in at least one individual in the ExAC database. These 330 genes were used as a negative control of HI genes.

### Non-neurodegeneration-or-neurodevelopment diseases-related genes

We accessed 1,189 genes related to non-mental-health diseases from Krishnan et al. (2016). These genes were identified from OMIM and used as negative genes for autism spectrum disorder-related genes. We reviewed these 1,189 genes and then excluded the genes related to neurodegenerative and/or neurodevelopmental diseases, including Alzheimer’s disease, Parkinson’s disease, Huntington’s disease, SA, multiple system atrophy, epilepsy, autism spectrum disorder, and schizophrenia. We retained 1,113 genes related to non-neurodegeneration-or-neurodevelopment diseases (NNN) as another negative control of HI genes (Supplementary Table 6).

### Data and code availability

Genes related to ALS, HMN, SA, SPG, and SMA are listed in Supplementary Tables 1–3. Single-cell transcriptome datasets are listed in Supplementary Table 4. HI genes, LoFT genes, and NNN-related genes are listed in Supplementary Table 6.

The code for investigating cell-type specificity and gene expression strictness is written in R-program and is released at GitHub.^3^

### Ethics approval

This study was reviewed and approved by the Ethics Review Committee at Hebei Medical University and was performed at Hebei Industrial Technology Research Institute of Genomics. No participant or donor was involved in our study.

## Results

### The involvement of motor neurons in ALS

**FIGURE 1:**
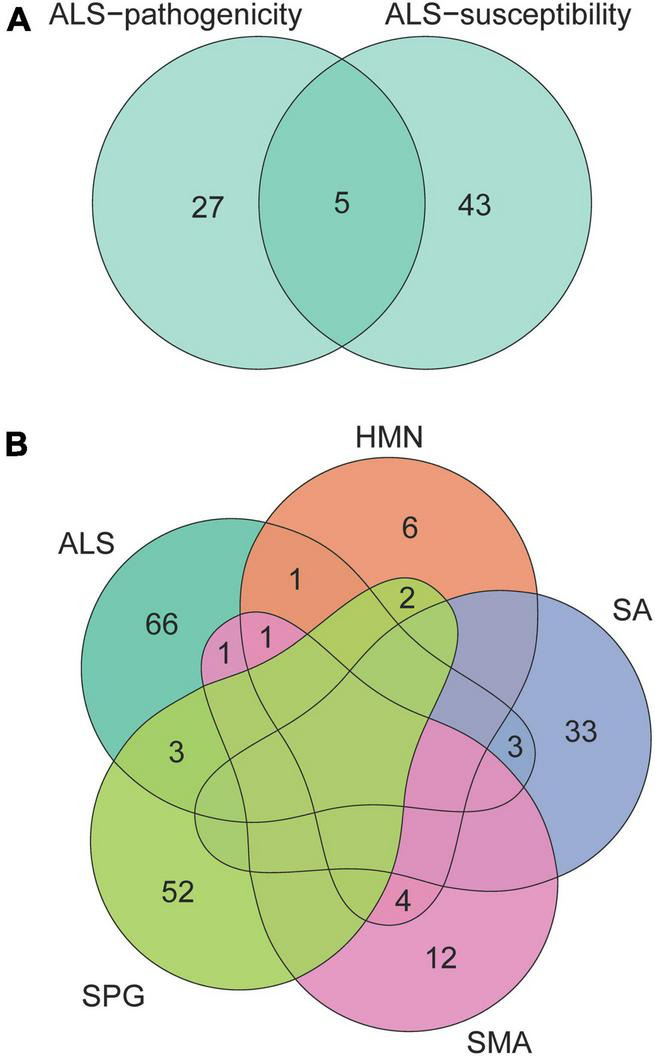
Venn diagram of genes related to ALS, HMN, SA, SPG, and SMA. The numbers in the area show the gene number in the corresponding overlap of genes related to ALS-pathogenicity and ALS-susceptibility **(A)**, genes related to ALS, HMN, SA, SPG, and SMA **(B)**. These neurological disorders with complex phenotypic overlaps share few genes.

**FIGURE 2:**
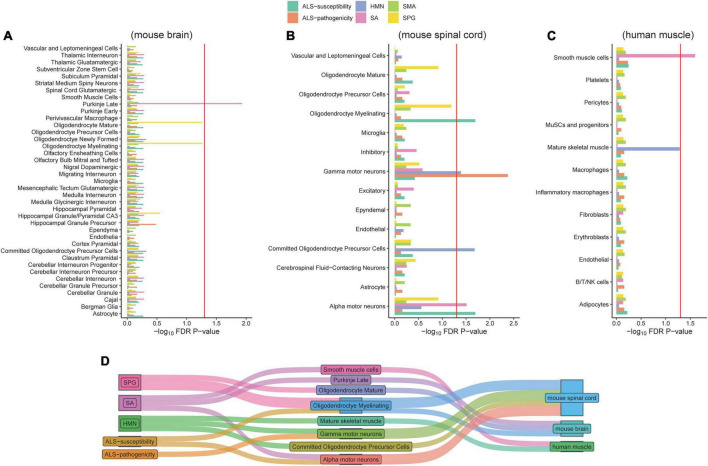
Associations of cell types and six gene panels. The red line refers to the significance threshold (*P*-adjusted <0.05) after FDR adjustment. Histograms exceeding the red line indicate the cell types of mouse brain **(A)**, mouse spinal cord **(B)**, and human muscle **(C)** significantly associated with corresponding gene lists. The Sankey diagram linked the diseases and associated cell types **(D)**.

We collected 32 ALS-pathogenicity genes from the OMIM database and 48 ALS-susceptibility genes from recent large-scale genome-wide association studies. There are five genes (*C9orf72*, *KIF5A*, *NEK1*, *SOD1*, and *TBK1*) related to the pathogenicity and susceptibility of ALS (Figure 1A). To address the cellular basis related to different genetic impacts, we employed the EWCE method developed by Skene and Grant (2016). The EWCE R-package was used to calculate the cell-type specificity of the pathogenicity and susceptibility ALS genes in the single-cell transcriptome data. No neurons in brain were found to be associated with ALS-pathogenicity genes or ALS-susceptibility genes (Figure 2A). Surprisingly, α-MNs and γ-MNs in the spinal cord were associated with ALS-susceptibility genes and ALS-pathogenicity genes (Figure 2B), suggesting different involvements of MNs subpopulations in ALS. To confirm our findings, we employed two independent single-cell transcriptome datasets of mouse spinal cord and annotated the cell types using the SingleR package with a reference from Rosenberg et al. (2018). We replicated the specific associations of susceptibility/pathogenicity genes and α-/γ- MNs (Supplementary Table 7). A significant association (*P*-value = 0.05) between susceptibility genes and α-MNs, as well as a significant association (*P*-value = 0.03) between pathogenicity genes and γ-MNs, was observed in the replication data of mouse spinal cord data (Milich’s dataset). Suggestive associations (*P*-value of susceptibility genes and α-MNs = 0.08; *P*-value of pathogenicity genes and γ-MNs = 0.075) were observed in the replication data of mouse spinal cord data (Sathyamurthy’s dataset). We also validated these associations in a single-cell transcriptome dataset of human spinal cord and observed a significant association (*P*-value = 0.039) between susceptibility genes and α-MNs, as well as a significant association (*P*-value = 0.015) between pathogenicity genes and γ-MNs. The replications in mouse dataset and validation in human dataset demonstrated the robustness of our findings. We combined the statistical summary of three independent single-cell transcriptome datasets of mouse spinal cord and one single-cell transcriptome dataset of human spinal cord *via* meta-analysis (Willer et al., 2010) and used the total number of cell types (56) of the four datasets [discovery: 14; replication of mouse spinal cord (Milich’s): 14; replication of mouse spinal cord data (Sathyamurthy’s): 10; validation of human spinal cord data: 18] for Bonferroni’s *P*-value correction. The meta-analysis results (Supplementary Table 7) confirmed γ-MNs associated with ALS-pathogenicity (*P*-value = 6.00 × 10^–7^) and α-MNs associated with ALS-susceptibility (*P*-value = 6.86 × 10^–6^).

### Cellular differences of ALS, HMN, SA, SPG, and SMA

Besides ALS, we included four neurological disorders—HMN, SA, SPG, and SMA—with phenotypic overlaps and collected their pathogenicity gene sets. Except for five of the HMN-pathogenicity genes shared with SMA, there are few overlaps between each pair among ALS, HMN, SA, SPG, and SMA (Figure 1B). These results showed potential different contributions of cell types to disease pathophysiology. Analysis of cell type-specific expression of genes related to HMN, SA, SPG, and SMA provided insights into their cellular basis: an association between Purkinje cells in brain and SA, an association between α-MNs in spinal cord and SA, an association between smooth muscle cells and SA, an association between γ-MNs in spinal cord and HMN, an association between oligodendrocyte and HMN, a suggestive association between mature skeletal muscle and HMN (*P*-value = 0.052), associations between oligodendrocyte subtypes and SPG (Figures 2A–C). The associations of MN/oligodendrocyte in spinal cord/brain were confirmed in replicated/validated datasets (Supplementary Table 8). Similar to ALS, MNs were associated with SA and HMN. To our knowledge, SA and HMN both present ALS-like phenotypes (Anand et al., 2014; Garcia-Santibanez et al., 2018) and MNs degeneration (Ikeda et al., 2012; Beijer and Baets, 2020) is involved in their pathogenesis. Oligodendrocyte is also involved in ALS, a cell type that was shown to induce hyperexcitability and death in mutant *SOD1* mouse (Ferraiuolo et al., 2016). Oligodendrocytes in both brain and spinal cord are associated with SPG, consistent with a previous report (Edgar et al., 2004). Oligodendrocytes are associated with HMN. Besides the cellular basis in brain and spinal cord, smooth muscle cells are associated with SA, skeletal muscle cells are associated with HMN. No evidence was observed for an association of SMA, even though SMA and HMN share five pathogenicity genes. To provide a clear image of the connections, we linked the diseases and cell types *via* a Sankey diagram (Figure 2D). These similarities and differences may provide insights in the etiology of ALS, HMN, SA, SMA, and SPG.

### The strict dosage requirement of ALS-susceptibility genes in MNs

**FIGURE 3:**
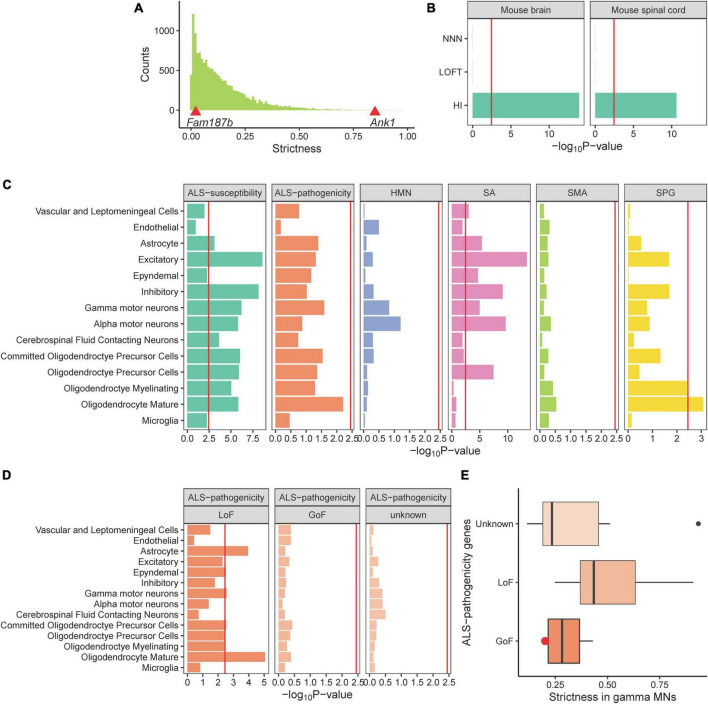
The strict dosage requirement of ALS-related genes in MNs. **(A)** The histogram displays the distribution of strictness value of all genes in brain cells. Strictness values for *Fam197b* and *Ank1* are exhibited by the red triangles. **(B)** Comparison of three gene panels with known strictness in mouse brain and spinal cord. **(C)** ALS-susceptibility genes dosage is required strictly in MNs. **(D,E)** LoF ALS-pathogenicity genes are required strictly in γ-MNs and no evidence are observed for GoF genes or unknown category. Red point refers to the *Sod1* gene.

Here we have revealed that ALS-related genes are specifically expressed in MNs, however, how these genes affect ALS reminds unclear. To provide insight into the mechanism, we developed a measure called strictness to evaluate the dosage requirement in single-cell populations. We hypothesized that if a gene is dosage-sensitive, it will have a narrow range of expression in normal cells and present a higher strictness measure. For example, *ANK1* was identified as a dosage-sensitive gene (Dang et al., 2008). The LoF variant in *ANK1* causes haploinsufficiency and results in autism spectrum disorder (Yang et al., 2019). The *Ank1* strictness measure in mouse brain cells is in the top strictness decile. In contrast, the *FAM187B* expression is tolerant to be altered because it harbors a common stop-gained variant (MacArthur et al., 2012). There are 43.6% of individuals who have a heterogeneous LoF variant and 34.7% of individuals who have homogeneous LoF variant in the gnomAD (Karczewski et al., 2020) database. The *Fam187b* strictness measure in mouse brain cells is in the bottom strictness decile (Figure 3A). To evaluate the performance of the strictness measure, we employed three gene sets with known expression patterns in the brain. Dosage-sensitive genes involved in brain functions are expected to have high strictness in brain cells. We collected 49 HI genes related to neurodegenerative diseases and/or neurodevelopmental diseases as a positive control, 300 LoFT genes, and 1,113 NNN disease-related genes as negative controls for the absence of dosage-sensitivity or brain functions, respectively. HI genes exhibited significant high strictness in brain and spinal cord. In contrast, LoFT genes and NNN disease-related genes exhibited low strictness (Figure 3B). These results demonstrated that strictness is a robust measure of dosage-sensitive genes.

Subsequently, we calculated the strictness of ALS-related genes in spinal cord cell types and showed that the ALS-susceptibility genes present significant high strictness in α-MNs (Figure 3C), indicating that the ALS-susceptibility genes are dosage-sensitive and the LoF of genes may be a mechanism of ALS. The *P*-value of the ALS-pathogenicity gene strictness in γ-MNs is close to the significant threshold, indicating that there may be different mechanism among these genes. We reviewed the ALS-pathogenicity genes on the basis of animal model and classified these genes into three categories: LoF, gain of function (GoF), and unknown (Supplementary Table 1). We showed that LoF ALS-pathogenicity genes are strictly required in γ-MNs. In contrast, no statistical evidence for supporting the hypothesis that the GoF genes or the genes in unknown category are dosage-sensitive (Figure 3D). These results are consistent with our hypothesis that LoF genes have high strictness and GoF genes have low strictness.

Besides, ALS-susceptibility genes and LoF pathogenicity genes exhibited high strictness in astrocytes and oligodendrocytes, both these two cell types were shown to play important roles in ALS (Ferraiuolo et al., 2016; Stoklund Dittlau et al., 2023). Excitatory, inhibitory, cerebrospinal fluid contacting neurons also required strict expressions of ALS-susceptibility genes. Abnormal cerebrospinal fluid contacting neurons (Ng Kee Kwong et al., 2020), imbalance between excitatory and inhibitory (Foerster et al., 2013; Cavarsan et al., 2022) were reported to contribute to ALS.

## Discussion

Our study revealed several cell types associated with ALS and three additional neurological disorders, HMN, SA, and SPG. Most of the related cell types have been demonstrated to be important in the related diseases (Skene et al., 2018; Bryois et al., 2020) [e.g., Purkinje cells (Kasumu and Bezprozvanny, 2012; Xia et al., 2013; Ishida et al., 2016) in SA, oligodendrocyte (Edgar et al., 2004) in SPG]. Notably, MNs are linked to ALS: α-MNs are associated with ALS-susceptibility genes and γ-MNs are associated with ALS-pathogenicity genes. Although the degeneration of MNs is demonstrated to cause ALS and neuromuscular disorders, the pathogenicity of MNs subpopulations is less known. The different roles of MNs subpopulations are consistent with a previous study (Lalancette-Hebert et al., 2016) that showed different vulnerabilities of MNs subpopulations: α-MNs are selectively degenerated and γ-MNs are completely spared in an ALS mutant mouse model. These results suggested that α- and γ-MNs do not play equal roles in ALS. Besides MNs, we found no statistical evidence for an association between muscle cells and ALS-related genes (Figure 2C). To our knowledge, MN death is the core event of ALS pathology (Anakor et al., 2022), however, the disruption of the neuromuscular junction is an early event in ALS pathology (Cappello and Francolini, 2017). Skeletal muscle metabolic dysregulation and atrophy in *SOD1* mutation transgenic mice (Brooks et al., 2004; Marcuzzo et al., 2011) and iPSCs (Badu-Mensah et al., 2020) derived from ALS patients harboring *SOD1* mutation were suggested to play a role in ALS. The muscle atrophy in *SOD1* model doesn’t conflict with our result. Our study investigated the most specific cell type expressed ALS-related genes. We cannot exclude the potential connection between specific gene and other cell types. Moreover, we observed *SOD1* is specifically expressed in human muscle (Supplementary Table 9). Limited to the power for detecting minor characteristics of a few genes, the *P*-value of gene expression specificity in muscle did not access the significant threshold after FDR. Taken together, these results deepened our understanding of ALS pathogenesis.

We showed that the ALS-susceptibility genes are dosage-sensitive in MNs, as well as the ALS-pathogenicity genes with known LoF mechanism in γ-MNs. In contrast, the GoF ALS-pathogenicity genes or the genes with unknown mechanism exhibited low strictness (Figures 3D, E). *SOD1* is one of the GoF pathogenicity genes. Transgenic mutant *SOD1* mice and rats develop characteristics that are similar to human ALS. A previous study showed that the complete absence of *SOD1* in mice did not precipitate ALS-related phenotypes (Reaume et al., 1996). A low strictness value of *Sod1* is consistent with the GoF mechanism (Figure 3E). Indeed, which mechanism causes the ALS—gain- or LoF—is still not clear (Kabashi et al., 2010; Saccon et al., 2013; Mizielinska and Isaacs, 2014; Scekic-Zahirovic et al., 2016). Our study suggested the main characteristics of ALS-susceptibility genes is dosage-sensitive, highlighting the need to carefully consider the LoF mechanism in sporadic ALS. For LoF ALS-pathogenicity genes, they were shown to strictly express in γ-MNs but not α-MNs. This result may suggest γ-MNs are more vulnerable to dosage alteration of the LoF pathogenicity genes. We noticed that there is no significant difference in the vulnerability of α- and γ-MNs to ALS-susceptibility genes. This result doesn’t conflict with the association between ALS-susceptibility genes and α-MNs. The ALS-susceptibility genes were shown to highly express in α-MNs. Compared with other cells with lower expression, the α-MNs were largely affected by the alteration of ALS-susceptibility genes. In contrast, the strictness of SPG-related genes was shown differences between oligodendrocytes and other un-associated cells, as well as that of ALS-pathogenicity genes and γ-MNs. Indeed, the strictness measure was designed to compare the different expression spectrums in specific cell type between LoF genes and GoF genes as a fellow independent study of cell type expression specificity analysis. This measure was demonstrated by the LoF ALS-pathogenicity genes strictly expressed in related γ-MNs and exhibited the strict expression of SPG-related genes in oligodendrocytes, SA-related genes in α-MNs, ALS-related genes in oligodendrocytes. HMN-related genes were shown no LoF characteristic in related MNs. Based on these results, strictness analysis may provide *a priori* understanding of the mechanisms of disease-related genes without an animal model.

Similar to ALS, MNs were associated with SA and HMN. To our knowledge, SA and HMN both present ALS-like phenotypes (Anand et al., 2014; Garcia-Santibanez et al., 2018), and MNs degeneration (Ikeda et al., 2012; Beijer and Baets, 2020) are involved in their pathogenesis. Oligodendrocytes are associated with ALS, SPG, and HMN. The links to SPG and ALS are consistent with previous reports (Edgar et al., 2004; Ferraiuolo et al., 2016). Besides, smooth muscle cells are associated with SA, and skeletal muscle cells are associated with HMN. No evidence was observed for an association of SMA, even though SMA and HMN share five pathogenicity genes. These similarities and differences may provide insights into the etiology of ALS, HMN, SA, SMA, and SPG.

Upper MNs should be considered in our further investigation. ALS patients showed loss of pyramidal tract upper MNs, specifically Betz cells (Hammer et al., 1979). The cortical connections of Betz cells are impaired prior to ALS onset (Genç et al., 2017). Betz cells were found below the surface of the cerebral cortex within layer V of the primary motor cortex and make direct connections to spinal MNs (Lemon, 2008). However, the mouse brain single-cell transcriptome datasets employed in our study did not annotate Betz cells. Betz cell specifically expressed *POU3F1* gene (Machado et al., 2014) which can be used as a marker for cell identification in mouse and human. A recent study with more than 450,000 transcriptomes and epigenomes in humans, marmoset monkeys and mice showed a broadly conserved cellular makeup of primary motor cortex, with similarities that mirror evolutionary distance and are consistent between the transcriptome and epigenome. Our discoveries in mouse spinal cord were validated in human spinal cord. These consistent results indicated data from mouse can be used for human disease investigation, providing a reliable pathway for further investigation in Betz cells and other novel cell types.

In summary, our study revealed the cellular basis of ALS, HMN, SA, and SPG and the dosage characteristic of the disease-related genes in specific cell types. Moreover, the methods—cell type enrichment and gene expression variability—are useful for investigating the role of genes in various cell types, which may open the door for valuable mining of public single-cell data and genetic knowledge of human diseases.

## Data availability statement

The original contributions presented in this study are included in the article/Supplementary material, further inquiries can be directed to the corresponding authors.

## Author contributions

HL and LG conceived the study. JZ, YL, and ZZ supervised the study. LG and MD collected the disease-related genes. HL performed the investigation and wrote the first draft of the manuscript. MD and ZZ confirmed the study design and research findings for ALS and other neurological disorders. HL, MD, KK, JZ, YL, and ZZ reviewed the manuscript. HL, LG, LB, KK, YL, JZ, and ZZ revised the manuscript. LB, KK, YL, and JZ contributed to a significant improvement of the manuscript. All authors contributed to the article and approved the submitted version.

## Footnotes

1
https://github.com/LTLA/SingleR

2
https://github.com/NathanSkene/EWCE

3
https://github.com/liuhankui/ALS

## References

[B1] Al-Chalabi A. Fang F. Hanby M. Leigh P. Shaw C. Ye W. (2010). An estimate of amyotrophic lateral sclerosis heritability using twin data. *J. Neurol. Neurosurg. Psychiatry* 81 1324–1326. 20861059 10.1136/jnnp.2010.207464 PMC2988617

[B2] Amberger J. S. Bocchini C. A. Schiettecatte F. Scott A. F. Hamosh A. (2015). OMIM. org: Online mendelian inheritance in man (OMIM§), an online catalog of human genes and genetic disorders. *Nucleic Acids Res.* 43 D789–D798. 10.1093/nar/gku1205 25428349 PMC4383985

[B3] Anakor E. Duddy W. J. Duguez S. (2022). The cellular and molecular signature of ALS in muscle. *J. Pers. Med.* 12:1868. 10.3390/jpm12111868 36579600 PMC9692882

[B4] Anand K. S. Wadhwa A. Garg J. Mahajan R. K. (2014). Amyotrophic lateral sclerosis-like presentation in a HIV-positive patient. *J. Int. Assoc. Providers AIDS Care (JIAPAC)* 13 515–518. 10.1177/2325957414535254 24842949

[B5] Andersen J. Thom N. Shadrach J. Chen X. Amin N. Yoon S. (2021). Landscape of human spinal cord cell type diversity at midgestation. *bioRxiv* [Preprint]. 10.1101/2021.12.29.473693

[B6] Aran D. Looney A. Liu L. Wu E. Fong V. Hsu A. (2019). Reference-based analysis of lung single-cell sequencing reveals a transitional profibrotic macrophage. *Nat. Immunol.* 20 163–172. 10.1038/s41590-018-0276-y 30643263 PMC6340744

[B7] Badu-Mensah A. Guo X. McAleer C. W. Rumsey J. W. Hickman J. J. (2020). Functional skeletal muscle model derived from SOD1-mutant ALS patient iPSCs recapitulates hallmarks of disease progression. *Sci. Rep.* 10:14302. 10.1038/s41598-020-70510-3 32868812 PMC7459299

[B8] Beijer D. Baets J. (2020). The expanding genetic landscape of hereditary motor neuropathies. *Brain* 143 3540–3563. 10.1093/brain/awaa311 33210134

[B9] Benyamin B. He J. Zhao Q. Gratten J. Garton F. Leo P. (2017). Cross-ethnic meta-analysis identifies association of the GPX3-TNIP1 locus with amyotrophic lateral sclerosis. *Nat. Commun.* 8 1–7. 10.1038/s41467-017-00471-1 28931804 PMC5606989

[B10] Brooks K. J. Hill M. D. W. Hockings P. D. Reid D. G. (2004). MRI detects early hindlimb muscle atrophy in Gly93Ala superoxide dismutase-1 (G93A SOD1) transgenic mice, an animal model of familial amyotrophic lateral sclerosis. *NMR Biomed.* 17 28–32. 10.1002/nbm.861 15011248

[B11] Brown R. H. Al-Chalabi A. (2017). Amyotrophic lateral sclerosis. *N. Engl. J. Med.* 377 162–172. 28700839 10.1056/NEJMra1603471

[B12] Bryois J. Skene N. Hansen T. Kogelman L. Watson H. Liu Z. (2020). Genetic identification of cell types underlying brain complex traits yields insights into the etiology of Parkinson’s disease. *Nat. Genet.* 52 482–493. 10.1038/s41588-020-0610-9 32341526 PMC7930801

[B13] Burke R. E. Strick P. L. Kanda K. Kim C. C. Walmsley B. (1977). Anatomy of medial gastrocnemius and soleus motor nuclei in cat spinal cord. *J. Neurophysiol.* 40 667–680. 874534 10.1152/jn.1977.40.3.667

[B14] Cappello V. Francolini M. (2017). Neuromuscular junction dismantling in amyotrophic lateral sclerosis. *Int. J. Mol. Sci.* 18 2092. 10.3390/ijms18102092 28972545 PMC5666774

[B15] Cavarsan C. Steele P. Genry L. Reedich E. McCane L. LaPre K. (2022). Inhibitory interneurons show early dysfunction in a SOD1 mouse model of amyotrophic lateral sclerosis. *J. Physiol.* 601 647–667. 10.1113/JP284192 36515374 PMC9898203

[B16] Dang V. T. Kassahn K. S. Marcos A. E. Ragan M. A. (2008). Identification of human haploinsufficient genes and their genomic proximity to segmental duplications. *Eur. J. Hum. Genet.* 16 1350–1357. 10.1038/ejhg.2008.111 18523451

[B17] De Micheli A. J. Spector J. A. Elemento O. Cosgrove B. D. (2020). A reference single-cell transcriptomic atlas of human skeletal muscle tissue reveals bifurcated muscle stem cell populations. *Skeletal Muscle* 10 1–13. 10.1186/s13395-020-00236-3 32624006 PMC7336639

[B18] Deng M. Wei L. Zuo X. Tian Y. Xie F. Hu P. (2013). Genome-wide association analyses in Han Chinese identify two new susceptibility loci for amyotrophic lateral sclerosis. *Nat. Genet.* 45 697–700. 10.1038/ng.2627 23624525

[B19] Dueck H. Khaladkar M. Kim T. Spaethling J. Francis C. Suresh S. (2015). Deep sequencing reveals cell-type-specific patterns of single-cell transcriptome variation. *Genome Biol.* 16 1–17. 26056000 10.1186/s13059-015-0683-4 PMC4480509

[B20] Edgar J. McLaughlin M. Yool D. Zhang S. Fowler J. Montague P. (2004). Oligodendroglial modulation of fast axonal transport in a mouse model of hereditary spastic paraplegia. *J. Cell Biol.* 166 121–131. 10.1083/jcb.200312012 15226307 PMC2172145

[B21] Ferraiuolo L. Meyer K. Sherwood T. Vick J. Likhite S. Frakes A. (2016). Oligodendrocytes contribute to motor neuron death in ALS via SOD1-dependent mechanism. *Proc. Natl. Acad. Sci. U.S.A.* 113 E6496–E6505. 10.1073/pnas.1607496113 27688759 PMC5081600

[B22] Foerster B. Pomper M. Callaghan B. Petrou M. Edden R. Mohamed M. (2013). An imbalance between excitatory and inhibitory neurotransmitters in amyotrophic lateral sclerosis revealed by use of 3-T proton magnetic resonance spectroscopy. *JAMA Neurol.* 70 1009–1016. 10.1001/jamaneurol.2013.234 23797905 PMC4382938

[B23] Fogh I. Ratti A. Gellera C. Lin K. Tiloca C. Moskvina V. (2014). A genome-wide association meta-analysis identifies a novel locus at 17q11. 2 associated with sporadic amyotrophic lateral sclerosis. *Hum. Mol. Genet.* 23 2220–2231. 10.1093/hmg/ddt587 24256812 PMC3959809

[B24] Garcia-Santibanez R. Burford M. Bucelli R. C. (2018). Hereditary motor neuropathies and amyotrophic lateral sclerosis: A molecular and clinical update. *Curr. Neurol. Neurosci. Rep.* 18 1–10. 30328519 10.1007/s11910-018-0901-z

[B25] Genç B. Jara J. Lagrimas A. Pytel P. Roos R. Mesulam M. (2017). Apical dendrite degeneration, a novel cellular pathology for Betz cells in ALS. *Sci. Rep.* 7:41765. 10.1038/srep41765 PMC5292972 28165465

[B26] Graham A. J. Macdonald A. M. Hawkes C. H. (1997). British motor neuron disease twin study. *J. Neurol. Neurosurg. Psychiatry* 62 562–569. 10.1136/jnnp.62.6.562 9219739 PMC1074137

[B27] Hammer R. P. J. Tomiyasu U. Scheibel A. B. (1979). Degeneration of the human Betz cell due to amyotrophic lateral sclerosis. *Exp. Neurol.* 63 336–346. 437007 10.1016/0014-4886(79)90129-8

[B28] Iacoangeli A. Lin T. Al Khleifat A. Jones A. Opie-Martin S. Coleman J. (2020). Genome-wide meta-analysis finds the ACSL5-ZDHHC6 locus is associated with ALS and links weight loss to the disease genetics. *Cell Rep.* 33:108323. 10.1016/j.celrep.2020.108323 33113361 PMC7610013

[B29] Ikeda Y. Ohta Y. Kobayashi H. Okamoto M. Takamatsu K. Ota T. (2012). Clinical features of SCA36: A novel spinocerebellar ataxia with motor neuron involvement (Asidan). *Neurology* 79 333–341. 22744658 10.1212/WNL.0b013e318260436f

[B30] Ishida Y. Kawakami H. Kitajima H. Nishiyama A. Sasai Y. Inoue H. (2016). Vulnerability of Purkinje cells generated from spinocerebellar ataxia type 6 patient-derived iPSCs. *Cell Rep.* 17 1482–1490. 27806289 10.1016/j.celrep.2016.10.026

[B31] Kabashi E. Lin L. Tradewell M. Dion P. Bercier V. Bourgouin P. (2010). Gain and loss of function of ALS-related mutations of TARDBP (TDP-43) cause motor deficits in vivo. *Hum. Mol. Genet.* 19 671–683. 10.1093/hmg/ddp534 19959528

[B32] Karczewski K. Francioli L. Tiao G. Cummings B. Alföldi J. Wang Q. (2020). The mutational constraint spectrum quantified from variation in 141,456 humans. *Nature* 581 434–443. 32461654 10.1038/s41586-020-2308-7 PMC7334197

[B33] Kasumu A. Bezprozvanny I. (2012). Deranged calcium signaling in Purkinje cells and pathogenesis in spinocerebellar ataxia 2 (SCA2) and other ataxias. *Cerebellum* 11 630–639. 20480274 10.1007/s12311-010-0182-9 PMC3257360

[B34] Kiernan M. Vucic S. Cheah B. Turner M. Eisen A. Hardiman O. (2011). Amyotrophic lateral sclerosis. *Lancet* 377 942–955. 21296405 10.1016/S0140-6736(10)61156-7

[B35] Krishnan A. Zhang R. Yao V. Theesfeld C. Wong A. Tadych A. (2016). Genome-wide prediction and functional characterization of the genetic basis of autism spectrum disorder. *Nat. Neurosci.* 19 1454–1462. 27479844 10.1038/nn.4353 PMC5803797

[B36] Laaksovirta H. Peuralinna T. Schymick J. Scholz S. Lai S. Myllykangas L. (2010). Chromosome 9p21 in amyotrophic lateral sclerosis in Finland: A genome-wide association study. *Lancet Neurol.* 9 978–985. 20801718 10.1016/S1474-4422(10)70184-8 PMC2965392

[B37] Lalancette-Hebert M. Sharma A. Lyashchenko A. K. Shneider N. A. (2016). Gamma motor neurons survive and exacerbate alpha motor neuron degeneration in ALS. *Proc. Natl. Acad. Sci. U.S.A.* 113 E8316–E8325. 10.1073/pnas.1605210113 27930290 PMC5187676

[B38] Lek M. Karczewski K. Minikel E. Samocha K. Banks E. Fennell T. (2016). Analysis of protein-coding genetic variation in 60,706 humans. *Nature* 536 285–291. 27535533 10.1038/nature19057 PMC5018207

[B39] Lemon R. N. (2008). Descending pathways in motor control. *Annu. Rev. Neurosci.* 31 195–218. 18558853 10.1146/annurev.neuro.31.060407.125547

[B40] Li C. Ou R. Wei Q. Shang H. (2021). Shared genetic links between amyotrophic lateral sclerosis and obesity-related traits: A genome-wide association study. *Neurobiol. Aging* 102 211.e1–211.e9. 10.1016/j.neurobiolaging.2021.01.023 33640203

[B41] Liu H.-K. He S.-J. Zhang J.-G. (2021). A bioinformatic study revealed serotonergic neurons are involved in the etiology and therapygenetics of anxiety disorders. *Transl. Psychiatry* 11 1–6. 10.1038/s41398-021-01432-5 34011923 PMC8134630

[B42] MacArthur D. G. Balasubramanian S. Frankish A. Huang N. Morris J. Walter K. (2012). A systematic survey of loss-of-function variants in human protein-coding genes. *Science* 335 823–828. 10.1126/science.1215040 22344438 PMC3299548

[B43] Machado C. B. Kanning K. Kreis P. Stevenson D. Crossley M. Nowak M. (2014). Reconstruction of phrenic neuron identity in embryonic stem cell-derived motor neurons. *Development (Cambridge, England)* 141 784–794. 10.1242/dev.097188 24496616 PMC3912827

[B44] Marcuzzo S. Zucca I. Mastropietro A. de Rosbo N. Cavalcante P. Tartari S. (2011). Hind limb muscle atrophy precedes cerebral neuronal degeneration in G93A-SOD1 mouse model of amyotrophic lateral sclerosis: A longitudinal MRI study. *Exp. Neurol.* 231 30–37. 10.1016/j.expneurol.2011.05.007 21620832

[B45] Marin B. Hamidou B. Couratier P. Nicol M. Delzor A. Raymondeau M. (2014). Population-based epidemiology of amyotrophic lateral sclerosis (ALS) in an ageing Europe–the French register of ALS in Limousin (FRAL im register). *Eur. J. Neurol.* 21 1292–300, e78–79. 10.1111/ene.12474 24909935

[B46] Mehta P. Raymond J. Punjani R. Larson T. Han M. Bove F. (2022). Incidence of amyotrophic lateral sclerosis in the United States, 2014-2016. *Amyotroph. Lateral Scler. Frontotemporal Degener.* 23 378–382. 10.1080/21678421.2021.2023190 35023792

[B47] Milich L. Choi J. Ryan C. Cerqueira S. Benavides S. Yahn S. (2021). Single-cell analysis of the cellular heterogeneity and interactions in the injured mouse spinal cord. *J. Exp. Med.* 218:e20210040. 10.1084/jem.20210040 34132743 PMC8212781

[B48] Mizielinska S. Isaacs A. M. (2014). C9orf72 amyotrophic lateral sclerosis and frontotemporal dementia: Gain or loss of function? *Curr. Opin. Neurol.* 27:515. 10.1097/WCO.0000000000000130 PMC4165481 25188012

[B49] Nakamura R. Misawa K. Tohnai G. Nakatochi M. Furuhashi S. Atsuta N. (2020). A multi-ethnic meta-analysis identifies novel genes, including ACSL5, associated with amyotrophic lateral sclerosis. *Commun. Biol.* 3:526. 10.1038/s42003-020-01251-2 32968195 PMC7511394

[B50] Ng Kee Kwong K. C. Mehta A. R. Nedergaard M. Chandran S. (2020). Defining novel functions for cerebrospinal fluid in ALS pathophysiology. *Acta Neuropathol. Commun.* 8:140. 10.1186/s40478-020-01018-0 32819425 PMC7439665

[B51] Nicolas A. Kenna K. Renton A. Ticozzi N. Faghri F. Chia R. (2018). Genome-wide analyses identify KIF5A as a novel ALS gene. *Neuron* 97 1268–1283. 10.1016/j.neuron.2018.02.027 29566793 PMC5867896

[B52] Ragagnin A. M. G. Shadfar S. Vidal M. Jamali M. S. Atkin J. D. (2019). Motor neuron susceptibility in ALS/FTD. *Front. Neurosci.* 13:532. 10.3389/fnins.2019.00532 31316328 PMC6610326

[B53] Reaume A. Elliott J. Hoffman E. Kowall N. Ferrante R. Siwek D. (1996). Motor neurons in Cu/Zn superoxide dismutase-deficient mice develop normally but exhibit enhanced cell death after axonal injury. *Nat. Genet.* 13 43–47. 10.1038/ng0596-43 8673102

[B54] Renton A. E. Chiò A. Traynor B. J. (2014). State of play in amyotrophic lateral sclerosis genetics. *Nat. Neurosci.* 17 17–23. 24369373 10.1038/nn.3584 PMC4544832

[B55] Rosenberg A. Roco C. Muscat R. Kuchina A. Sample P. Yao Z. (2018). Single-cell profiling of the developing mouse brain and spinal cord with split-pool barcoding. *Science* 360 176–182. 10.1126/science.aam8999 29545511 PMC7643870

[B56] Saccon R. A. Bunton-Stasyshyn R. K. A. Fisher E. M. C. Fratta P. (2013). Is SOD1 loss of function involved in amyotrophic lateral sclerosis? *Brain* 136 2342–2358. 23687121 10.1093/brain/awt097 PMC3722346

[B57] Saraiva L. R. Ibarra-Soria X. Khan M. Omura M. Scialdone A. Mombaerts P. (2015). Hierarchical deconstruction of mouse olfactory sensory neurons: From whole mucosa to single-cell RNA-seq. *Sci. Rep.* 5 1–17. 10.1038/srep18178 26670777 PMC4680959

[B58] Sathyamurthy A. Johnson K. Matson K. Dobrott C. Li L. Ryba A. (2018). Massively parallel single nucleus transcriptional profiling defines spinal cord neurons and their activity during behavior. *Cell Rep.* 22 2216–2225. 10.1016/j.celrep.2018.02.003 29466745 PMC5849084

[B59] Scekic-Zahirovic J. Sendscheid O. El Oussini H. Jambeau M. Sun Y. Mersmann S. (2016). Toxic gain of function from mutant FUS protein is crucial to trigger cell autonomous motor neuron loss. *EMBO J.* 35 1077–1097. 10.15252/embj.201592559 26951610 PMC4868956

[B60] Shatunov A. Mok K. Newhouse S. Weale M. Smith B. Vance C. (2010). Chromosome 9p21 in sporadic amyotrophic lateral sclerosis in the UK and seven other countries: A genome-wide association study. *Lancet Neurol.* 9 986–994. 10.1016/S1474-4422(10)70197-6 20801717 PMC3257853

[B61] Skene N. G. Grant S. G. N. (2016). Identification of vulnerable cell types in major brain disorders using single cell transcriptomes and expression weighted cell type enrichment. *Front. Neurosci.* 10:16. 10.3389/fnins.2016.00016 26858593 PMC4730103

[B62] Skene N. Bryois J. Bakken T. Breen G. Crowley J. Gaspar H. (2018). Genetic identification of brain cell types underlying schizophrenia. *Nat. Genet.* 50 825–833. 29785013 10.1038/s41588-018-0129-5 PMC6477180

[B63] Stoklund Dittlau K. Terrie L. Baatsen P. Kerstens A. De Swert L. Janky R. (2023). FUS-ALS hiPSC-derived astrocytes impair human motor units through both gain-of-toxicity and loss-of-support mechanisms. *Mol. Neurodegener.* 18:5. 10.1186/s13024-022-00591-3 36653804 PMC9847053

[B64] Usoskin D. Furlan A. Islam S. Abdo H. Lönnerberg P. Lou D. (2015). Unbiased classification of sensory neuron types by large-scale single-cell RNA sequencing. *Nat. Neurosci.* 18 145–153. 10.1038/nn.3881 25420068

[B65] van Es M. Hardiman O. Chio A. Al-Chalabi A. Pasterkamp R. Veldink J. (2017). Amyotrophic lateral sclerosis. *Lancet* 390 2084–2098. 28552366 10.1016/S0140-6736(17)31287-4

[B66] van Rheenen W. Shatunov A. Dekker A. McLaughlin R. Diekstra F. Pulit S. (2016). Genome-wide association analyses identify new risk variants and the genetic architecture of amyotrophic lateral sclerosis. *Nat. Genet.* 48 1043–1048. 27455348 10.1038/ng.3622 PMC5556360

[B67] van Rheenen W. van der Spek R. Bakker M. van Vugt J. Hop P. Zwamborn R. (2021). Common and rare variant association analyses in amyotrophic lateral sclerosis identify 15 risk loci with distinct genetic architectures and neuron-specific biology. *Nat. Genet.* 53 1636–1648. 34873335 10.1038/s41588-021-00973-1 PMC8648564

[B68] Wei L. Tian Y. Chen Y. Wei Q. Chen F. Cao B. (2019). Identification of TYW3/CRYZ and FGD4 as susceptibility genes for amyotrophic lateral sclerosis. *Neurol. Genet.* 5:e375. 10.1212/NXG.0000000000000375 31872054 PMC6878836

[B69] Willer C. J. Li Y. Abecasis G. R. (2010). METAL: Fast and efficient meta-analysis of genomewide association scans. *Bioinformatics (Oxford, England)* 26 2190–2191. 10.1093/bioinformatics/btq340 20616382 PMC2922887

[B70] Xia G. McFarland K. Wang K. Sarkar P. Yachnis A. Ashizawa T. (2013). Purkinje cell loss is the major brain pathology of spinocerebellar ataxia type 10. *J. Neurol. Neurosurg. Psychiatry* 84 1409–1411. 10.1136/jnnp-2013-305080 23813740 PMC3923576

[B71] Xie T. Deng L. Mei P. Zhou Y. Wang B. Zhang J. (2014). A genome-wide association study combining pathway analysis for typical sporadic amyotrophic lateral sclerosis in Chinese Han populations. *Neurobiol. Aging* 35 1778.e9–1778.e23. 10.1016/j.neurobiolaging.2014.01.014 24529757

[B72] Xu L. Chen L. Wang S. Feng J. Liu L. Liu G. (2020). Incidence and prevalence of amyotrophic lateral sclerosis in urban China: A national population-based study. *J. Neurol. Neurosurg. Psychiatry* 91 520–525. 10.1136/jnnp-2019-322317 32139654

[B73] Yang R. Walder-Christensen K. Kim N. Wu D. Lorenzo D. Badea A. (2019). ANK2 autism mutation targeting giant ankyrin-B promotes axon branching and ectopic connectivity. *Proc. Natl. Acad. Sci. U.S.A.* 116 15262–15271. 10.1073/pnas.1904348116 31285321 PMC6660793

[B74] Zeisel A. Muñoz-Manchado A. Codeluppi S. Lönnerberg P. La Manno G. Juréus A. (2015). Brain structure. Cell types in the mouse cortex and hippocampus revealed by single-cell RNA-seq. *Science* 347 1138–1142. 25700174 10.1126/science.aaa1934

